# Staff perspectives on using long-acting antiretroviral treatment among persons being released from carceral settings in Baltimore, Maryland, USA

**DOI:** 10.1080/09540121.2025.2562459

**Published:** 2025-10-08

**Authors:** Zaire Cullins, Aurielle Thomas, Curt G. Beckwith, Michael Gordon, Thomas Blue, Christian Black, Hannah Camp, Lauren Brinkley-Rubinstein

**Affiliations:** a Department of Population Health Sciences, Duke University, Durham, NC, USA; b Division of Infectious Diseases, The Miriam Hospital, Providence, RI, USA; c Warren Alpert Medical School, Brown University, Providence, RI, USA; d Friends Research Institute, Inc., Baltimore, MD, USA; e Center for Environmental Health Disparities, University of California, Irvine, Irvine, CA, USA

**Keywords:** Long acting injectables, criminal legal system, HIV treatment, SDG 3: Good health and well-being, SDG 10: Reduced inequalities

## Abstract

The prevalence of HIV among individuals who experience incarceration is estimated to be three times higher than that of the general population in the United States. Currently, the standard of care, antiretroviral medications taken daily, is effective in reducing mortality and HIV transmission but individuals experiencing community re-entry face barriers while re-integrating with their community which may make adherence to daily medication challenging. Long acting injectable antiretroviral treatment (LAI ART) may offer an opportunity for individuals experiencing community re-entry to remain in treatment while prioritizing their other needs. Our team conducted 16 interviews with various staff in both community and carceral settings pertaining to the potential administration of LAI ART in prison settings with treatment continuing while individuals are experiencing community re-entry. Many participants were supportive of administering LAI ART in this population, but challenges related to ensuring continuation of care, access to clinics, and the lack of resources in health settings were frequently cited as potential barriers.

The prevalence of HIV among people who are incarcerated in the United States (US) is three times higher than that of the general population (and in any given year, 1 in 7 people with HIV are incarcerated in US jails or prisons) (Maruschak, 2021; Spaulding et al., 2009). The standard of care in both community and carceral settings for antiretroviral treatment (ART) for HIV is a combination of medications taken orally, often in a fixed dose combination pill taken once daily. Oral ART is effective in reducing mortality and HIV transmission but is contingent upon high levels of adherence (Nance et al., 2018). For individuals with HIV, the period of time following release from a carceral setting, known as community re-entry, is associated with decreased ART adherence and poor treatment outcomes (Dong et al., 2021; Ibañez et al., 2021; Pulitzer et al., 2021). Individuals tend to be released into chaotic environments that force them to confront competing needs such as reconnecting with loved ones, finding employment, connecting with community-based healthcare, obtaining housing, and often addressing mandated community supervision (Pulitzer et al., 2021; Rowell-Cunsolo et al., 2020). Faced with these competing needs, individuals may not remain adherent to their oral ART regimen and have challenges connecting with an HIV provider or refilling their prescriptions (Rowell-Cunsolo et al., 2020). To improve outcomes, many interventions to support ART adherence and linkage to HIV care after release from carceral settings have been developed but with limited success (Solomon & Sax, 2015).

Long-acting injectable (LAI) ART was developed as an alternative to oral medications to address the challenges of adherence (Markowitz and Meyers, 2015). LAI ART brings the hope of improved adherence, viral suppression, and improved long-term control of HIV among persons who have struggled with oral ART (Hickey et al., 2015). LAI ART has an advantage over daily oral medications by preventing sub-therapeutic drug levels that result from missed oral doses which can lead to treatment failure and drug resistance. The first FDA-approved LAI ART regimen includes an integrase inhibitor, cabotegravir (CAB), and a non-nucleoside reverse transcriptase inhibitor, rilpivirine (RPV), given as a combination every four or eight weeks. Injectable CAB/RPV was FDA-approved in January 2021 as a treatment regimen to replace an existing oral ART regimen among persons with viral suppression (NIH, 2021). Use of LAI ART has not been explored in carceral populations or for use during the community re-entry period in the US. In this study, we investigated staff perspectives on barriers and facilitators to using LAI ART during incarceration and continuing after release.

## Methods

Our team completed 16 virtual, one-hour interviews with administrative and medical staff from community healthcare settings and prison facilities in Maryland. The study team employed a purposive sampling method, inviting staff employed at relevant community and carceral organizations to participate in the study. Snowball sampling was also utilized, with some participants providing contact information for other individuals within their organization who had experience relevant to the interview topics. Of the 18 individuals contacted, one individual refused to participate and the other was a no-show to their rescheduled interview time. Participants completed one semi-structured interview addressing the following domains: (1) previous knowledge and experiences with LAI ART, (2) perceived barriers and facilitators of administering LAI ART in both community and carceral healthcare settings, (3) the perceived risks and benefits of utilizing LAI ART, and (4) perspectives on co-locating HIV treatment with medications for opioid use disorder (MOUD). All participants provided verbal informed consent to participate and to be audio-recorded. The study protocol was reviewed and approved by the institutional review boards at Duke University (Pro00112346). Audio recordings were transcribed by Rev.com and GMR Transcription services. A general inductive framework was utilized to create the codebook which was finalized using open and axial coding. Two research staff members independently coded each transcript in the qualitative analysis software, NVivo, and discrepancies were discussed and resolved amongst the larger research team.

## Results

Demographic information was collected from all participants prior to their interview. Among the 16 participants, 12 (75%) identified as women, 7 (43%) identified as Black, 6 (37%) identified as White, one participant identified as Hispanic or Latinx, one identified as Asian, and one identified as “Other” race/ethnicity. Nearly half of the participants (n = 7) worked in community healthcare settings, with the remaining participants providing either healthcare to individuals in prison (n = 3) or working in an administrative role for the public safety department (n = 6). Overall, most participants (69%) worked in administrative roles, four (25%) were physicians (three provided care inside carceral facilities and one provided care in a community setting), and one participant was a pharmacist in a community setting. On average, participants had been employed in their current roles for three years.

Following analysis of the interviews, several major themes emerged relating to the advantages and disadvantages of LAI ART and strategies to assist with adherence among this population. Most (n = 13) participants indicated having some familiarity of LAI ART. Only two participants reported prescribing LAI ART in the past. Most participants were in support of administering LAI ART in general, citing that “[LAI ART] is going to be a game changer,” for individuals in carceral populations and those planned for release.

### Advantages

Many participants highlighted the advantages of LAI ART focusing specifically on optimal timing intervals, which subpopulations would benefit the most, and potential benefits to staff.

### Monthly injections vs. daily oral medications

All participants discussed potential advantages of LAI over daily oral ART. One participant stated that administering LAI ART every month or every other month was “definitely an advantage”, with another adding “life gets complicated, and patients are more successful when they don’t have to think about taking something … every day.” This participant goes on to discuss how these timing intervals could be very beneficial for patients “coming out of incarcerated settings [who] may be having other challenges … they need to focus on … such as getting a job and getting reestablished.” Another participant cited LAI ART as helping patients with “privacy … confidentiality” as they would only have to attend a provider visit rather than carry around their pills, potentially decreasing HIV-associated stigma often encountered by persons with HIV as “Anybody can say that’s a doctor’s appointment, doesn’t have to know what it is about.”

### Specific populations

Some participants mentioned that LAI ART would be especially beneficial for specific populations such as individuals with other chronic conditions who may experience pill fatigue and unhoused individuals as adherence to medication may be difficult when other needs are prioritized above treatment. This is particularly relevant for individuals experiencing community re-entry as this population is more likely to have multiple chronic conditions and experience homelessness compared to the general population. One participant stated

Once they are released to the community, there are other priorities. So the Maslow’s Hierarchy, you need shelter, food, and then you can be able to address your health … So if you don’t have that basic needs, and with that change in environment when you’re coming out to the community, and you have to take care of so many things that Cabenuva … will be a great advantage for such patients.

For similar reasons, few participants voiced that individuals who are not yet virally suppressed could also benefit from LAI ART. One participant noted “Thus far, the medication has only been approved for people who were already virally suppressed, which I think we can all agree is not our target population to benefit from a long-acting injectable.”

### Staffing benefits

Many participants believed LAI ART has advantages for healthcare staff as well, with one participant noting that starting and/or continuing patients on LAI ART would be an advantage “Both for the patient and also for staff, so it means instead of giving daily medication, when you give once a month, it makes really big difference”. This point was especially salient for healthcare staff in carceral settings as some participants felt LAI ART could alleviate some of the frequently encountered challenges of understaffing, highlighted by a participant stating that, “[LAI ARTs are] going to eliminate an extra staffing issue right there because you don’t have a person have to come around and give them the medication, it’s already in there.”

### Disadvantages

Some participants discussed disadvantages related to LAI ART such as potential complications with continuing care during community re-entry, fears surrounding patients missing appointments, issues regarding patients’ access to clinics, and insufficient resourcing of health centers, both in carceral and community settings.

### Continuation of care

Though many participants believe that LAI ART is a valuable addition to the HIV treatment landscape, multiple concerns were voiced – the most common of which related to ensuring a continuation of care for individuals leaving prisons. For instance, one participant said: “If you start medication [during incarceration] and when they … go out and it’s very expensive or they don’t get it, no purpose to start then stop”. Most (n = 11) participants noted that issues of insurance coverage and cost were a big concern, “If you prescribe something and … try to go fill it … and it’s a large copay, even though we have assistance … that’s still pretty shocking for patients”. Additionally, participants in community settings noted that a warm hand-off between carceral and community staff is essential to ensure participants can maintain their adherence. A participant working in an administrative position in a community healthcare setting noted that their “medical case management team don’t have clearance to go inside the jail”, potentially inhibiting rapport-building between the community clinics and persons with HIV inside prison, which could impact adherence upon release. Several participants that worked in community settings that care for patients during the community re-entry period cited that the lack of release information between carceral and community providers poses a barrier for them – many of their patients aren’t aware of what their exact release date is and some don’t know where they are being released to, complicating the continuation of care. One participant expressed this by stating “We have to work a little bit closer with the prison system for that collaborative piece to help us because once they get discharged, sometimes they’re not where they say they’re going to be and then we can’t find them”; another participant illuminated this point by saying, “The only negative side I see from the re-entry perspective is that we may not know when they’re leaving and there being a gap.”

### Missed appointments

Some participants expressed fears surrounding patients’ ability to maintain adherence and potentially missing appointments. One participant stated this directly, mentioning that “The one caveat is that sometimes people forget that they have to go to the doctor.” Another participant stated that

Disadvantages we need to highlight to the patient that keeping their appointment is important. For any reason, if they have to – if they are not able to complete their treatment, if they’re not able to do their labs, if they’re not able to see their providers, it may cause harm to them rather than benefit.

### Access

Other concerns expressed by participants were surrounding access to the medication due to transportation barriers, with one participant noting

You know, Maryland in general – we have some areas that are very metropolitan, and we have some very rural areas so access to health care providers, access to public transportation in some of those more rural areas is a real challenge. So, for some people, that access to care in order to get the injectable could be hard.

### Resource needs

When it came to issues of ensuring both carceral and community settings would be able to administer this medication, participants noted multiple potential barriers. A few participants mentioned concerns regarding inadequate storage, as the injection needs to be refrigerated. One participant working as a doctor in a carceral setting noted that “they do not require medical grade refrigerators in prison facilities”, going on to say, “we do from time to time have refrigerators that’s going out of the temperature range and we have to waste a lot of medications”.

Many participants noted that additions to staffing would be necessary, both for community and carceral healthcare settings, to facilitate the administration of the injection, exemplified by one participant in the community stating that “nursing support is critical” to the administration of an LAI ART protocol. Several participants highlighted the need to educate nurses on the protocol, with one participant mentioning, “you would need training for the nursing staff as well” with another participant adding, “nursing support is critical”. One participant noted the importance of having staff who are able to educate and listen to patients, stating “I think sometimes our providers are doing medical care and that anything that involves counseling education tends to be extra … I think it’s imperative to have some type of a resource that can do that additional education”.

Additionally, several participants mentioned ongoing conversations regarding the classification of the injection as a medical benefit or a pharmacy benefit. This particularly resonated among some staff who worked in federally qualified health centers (FQHC) as

if I give it to them as a medical benefit because I’m an FQHC, I don’t get reimbursed the full cost of the medication. That visit is just covered under what they call a [prospective payment system] rate for us as FQHCs, which will hardly cover the cost of that medication.

### Adherence boost

When asked what services could be provided to patients to aid in their adherence to LAI ART, participants cited dedicated linkage to care teams, addressing social determinants of health, providing patients with education on LAI ART, and innovations relating to LAI ART access such as co-locating with other services, allowing pharmacists to administer or exploring self-administration methods.

Half of the participants supported having a care team that can support patients by offering services such as scheduling an appointment with a community HIV provider prior to leaving prison and providing case management services during community re-entry. One participant highlighted this by stating “You have the linkage to care team … You’re going to have to have somebody doing the telephone calls, doing the appointment reminders, scheduling their lab.” Another participant noted that “You need to have certain protocols … when they’re re-entered into the community, you need to have certain pharmacists, pharmacies where they can go and certain doctors to follow up on them. So there are no lapse in care”.

Relevant to social determinants of health, a majority of participants believed that providing transportation and housing would be beneficial to adherence in addition to other ancillary services such as employment and access to a telephone would also be beneficial. A participant highlighted the need to provide these services by stating,

I would say first and foremost is making sure we prioritize and address those social determinants, whether it’s arranging the transportation, whether it’s providing that job opportunity so they can know what they’re doing in regards to work, and employment, and housing. Make that easy, so that’s not something that they have to prioritize over their healthcare.

When it came to patient education, participants mentioned that providing patients with education relevant to safety and possible risk of resistance to HIV medications was necessary and important, demonstrated by one participant who said,

As long as the patient is educated and they know the advantages and disadvantages of the medication … A lot of the incarcerated population, they don’t have the information so that they can make an informed decision. So, it’s going to take … time from the providers, from the nurses, staffing to make sure that the patient is well educated.

When questioned about innovations that may help adherence to LAI ART, many participants were in support of co-locating other services, such as medications for opioid use disorder (MOUD), with one participant stating

[A]s much as you can couple the services together, I think the greater the benefit. And we know that those two things go together so very frequently for the population that looking at a one-stop-shop for as many of the services that are needed as possible.

A few participants were against diversifying the roles of those giving the injections, preferring leaving the administration of the injections in the hands of a medical provider (e.g., a nurse or a medical doctor), with one participant questioning “Why does it have to be different?” Other participants endorsed this possibility stating that, “I feel like the self-administration is probably the best bet if we can train individuals to be able to do that cleanly and safely and give them the tools.” Another participant voiced their support behind pharmacist administering this by mentioning “I mean, pharmacists administer vaccines, so why not?”

## Discussion

In this study, we examined staff understanding and perceptions of the use of LAI ART in carceral and community re-entry settings in the United States. Participants broadly supported the use of LAI ART for persons leaving prisons in Maryland. Participants highlighted what they viewed as the advantages and disadvantages of LAI ART and specifically highlighted ways in which adherence could be supported during the often chaotic community re-entry period. Overall, participants who interacted with persons with HIV who are incarcerated noted their strong support for LAI ART implementation prior to release from a carceral setting.

However, participants also voiced concerns surrounding patients’ ability to secure medication and keep appointments as issues such as insurance coverage, access to clinics, the social determinants of health and the importance of a warm handoff were prevalent throughout the interviews. To combat these challenges, participants expressed options such as exploring alternative administrators of the injection such as pharmacists or even self-administration, providing patients with housing, transportation, phones, assisting with insurance coverage, and co-locating other services at clinics that administer the injections.

There is very limited research on the use of LAI ART with carceral populations in the US, but recent studies have demonstrated that patients tend to prefer LAI ART over the daily oral ART given it is perceived as more convenient, allows people to eliminate pill burden, and may allow them to avoid stigma surrounding their HIV status (Collins et al., 2023; Fletcher et al., 2023, january 10, 2024; Kerrigan et al., 2018). Previous studies that documented stakeholder attitudes toward LAI ART in other settings also noted benefits to LAI ART while acknowledging that retention in care is necessary for patients (Koester et al., 2023). This sentiment was also expressed by participants in our study.

Taking these results into consideration, it becomes clear that LAI ART must be considered as one way to improve HIV treatment outcomes, in support of the Ending the HIV Epidemic (EHE) initiative. Additionally, several participants expressed that the potential of LAI ART could be great, even for populations that are not already virally suppressed on the oral regimen. Limited research investigating LAI ART in populations where individuals have viremia or experience challenges with adherence exists, with positive outcomes for individuals enrolled in these programs (Gandhi et al., 2023), underscoring the need for more research in this arena.

### Limitations

While this study is the first to investigate carceral and community staff attitudes surrounding LAI ART for individuals who are incarcerated and recently released individuals within the US, there are some limitations to our study. Study participants were recruited from Baltimore, MD, potentially rendering our findings less generalizable to other locations and correctional systems. Additionally, interviews were conducted mostly with administrative staff rather than providers, possibly decreasing representation of provider’s perspectives. Future studies need to investigate the perspectives of persons with HIV who are incarcerated or with a history of incarceration, to gain an understanding of how they view this treatment option inside carceral facilities and during community re-entry.

## Conclusion

LAI ART is a new HIV treatment option that may be preferable to daily oral ART regimens for eligible persons with HIV who experience incarceration. When interviewed, carceral and community staff stated that LAI ART has the potential to be a viable option for individuals recently released from prison in the United States. However, implementation of any future LAI ART program must address possible challenges related to social determinants of health in order to retain patients in their HIV care regimen. Though the medication could advance the goals of the EHE initiative currently, more research should be conducted investigating the use of LAI ART in populations who may struggle with adherence or individuals with viremia.
